# Evaluating the impact of adjunctive istradefylline on the cumulative dose of levodopa-containing medications in Parkinson’s disease: study protocol for the ISTRA ADJUST PD randomized, controlled study

**DOI:** 10.1186/s12883-022-02600-w

**Published:** 2022-03-03

**Authors:** Taku Hatano, Osamu Kano, Renpei Sengoku, Asako Yoritaka, Keisuke Suzuki, Noriko Nishikawa, Yohei Mukai, Kyoichi Nomura, Norihito Yoshida, Morinobu Seki, Miho Kawabe Matsukawa, Hiroo Terashi, Katsuo Kimura, Jun Tashiro, Shigeki Hirano, Hidetomo Murakami, Hideto Joki, Tsuyoshi Uchiyama, Hideki Shimura, Kotaro Ogaki, Jiro Fukae, Yoshio Tsuboi, Kazushi Takahashi, Toshimasa Yamamoto, Naotake Yanagisawa, Hiroshi Nagayama

**Affiliations:** 1 Department of Neurology, Faculty of Medicine, Juntendo University, 2-1-1 Hongo, Bunkyo-ku, 113-8421 Tokyo, Japan; 2 Department of Neurology, Faculty of Medicine, Toho University, 6-11-1 Omorinishi, Ota-ku, Tokyo, 143-8541 Japan; 3 Department of Neurology, Jikei University Daisan Hospital, 4-11-1 Izumihoncho, Komae, Tokyo 201-0003 Japan; 4 Department of Neurology, Juntendo University Koshigaya Hospital, 560 Fukuroyama, Koshigaya-shi, Saitama, 343-0032 Japan; 5 Department of Neurology, Dokkyo Medical University Hospital, 880 Oaza Kitakobayashi, Mibu-machi, Shimotsuga-gun, Tochigi, 321-0293 Japan; 6 Department of Neurology, National Center of Neurology and Psychiatry, 4-1-1 Ogawahigashi-cho, Kodaira-shi, Tokyo, 187-8551 Japan; 7 Department of Neurology, Saitama Medical Center, Kawagoe-shi, Saitama, 350-8550 Japan; 8 Department of Neurology, Keio University School of Medicine, 35 Shinanomachi, Shinjuku-ku, Tokyo, 160-8582 Japan; 9 Department of Neurology, Tokyo Metropolitan Geriatric Hospital and Institute of Gerontology, 35-2 Sakae-cho, Itabashi-ku, Tokyo, 173-0015 Japan; 10 Department of Neurology, Tokyo Medical University Hospital, 6-7-1 Nishi-shinjuku, Shinjuku-ku, Tokyo, 160-0023 Japan; 11 Department of Neurology, Yokohama City University Medical Center, 4-57 Urafune-cho, Minami-ku, Yokohama-shi, Kanagawa, 232-0024 Japan; 12 Sapporo Parkinson MS Neurological Clinic, Dai 27 Big Sapporo-kita Sky Building 12F, 7-6 Kita-7 jo Nishi-5 chome, Kita-ku, Sapporo-shi, Hokkaido, 060-0807 Japan; 13 Department of Neurology, Graduate School of Medicine, Chiba University, 1-8-1 Inohana, Chuo-ku, Chiba-shi, Chiba, 260-8670 Japan; 14 Department of Neurology, The Jikei University Hospital, 3-19-18 Nishishinbashi, Minato-ku, Tokyo, 105-8471 Japan; 15 Department of Neurology and Stroke Medicine, Yokohama City University Graduate School of Medicine, 3-9 Fukuura, Kanazawa-ku, Yokohama, 236-0004 Japan; 16 Department of Neurology, Seirei Hamamatsu General Hospital, 2-12-12 Sumiyoshi, Naka-ku, Hamamatsu-shi, Shizuoka, 430-8558 Japan; 17 Department of Neurology, Juntendo Tokyo Koto Geriatric Medical Center, 3-3-20 Shinsuna, Koto-ku, Tokyo, 136-0075 Japan; 18 Department of Neurology, Juntendo University Urayasu Hospital, 2-1-1 Tomioka, Urayasu-shi, Chiba, 279-0021 Japan; 19 Department of Neurology, Juntendo University Nerima Hospital, 3-1-10 Takano-dai, Nerima-ku, Tokyo, 177-8521 Japan; 20 Department of Neurology, Fukuoka University School of Medicine, 7-45-1 Nanakuma, Johnan-ku, Fukuoka, 814-0180 Japan; 21 Department of Neurology, Tokyo Metropolitan Neurological Hospital, Musashidai 2-6-1, Fuchu-shi, Tokyo, 183-0042 Japan; 22 Department of Neurology, Saitama Medical University Hospital, Saitama Medical University, 38 Morohongo, Moroyama-machi, Iruma-gun, Saitama, 350-0495 Japan; 23 Medical Technology Innovation Center, Juntendo University and Juntendo Clinical Research and Trial Center, 2-1-1 Hongo, Bunkyo-ku, Tokyo, 113-8421 Japan; 24 Department of Neurology, Nippon Medical School, 1-1-5 Sendagi, Bunkyo-ku, Tokyo, 113-8602 Japan

**Keywords:** Istradefylline, Parkinson’s disease, Adenosine A_2A_ receptor antagonist, Levodopa, Levodopa dose

## Abstract

**Background:**

Levodopa remains the most effective symptomatic treatment for Parkinson’s disease (PD) more than 50 years after its clinical introduction. However, the onset of motor complications can limit pharmacological intervention with levodopa, which can be a challenge when treating PD patients. Clinical data suggest using the lowest possible levodopa dose to balance the risk/benefit. Istradefylline, an adenosine A_2A_ receptor antagonist indicated as an adjunctive treatment to levodopa-containing preparations in PD patients experiencing wearing off, is currently available in Japan and the US. Preclinical and preliminary clinical data suggested that adjunctive istradefylline may provide sustained antiparkinsonian benefits without a levodopa dose increase; however, available data on the impact of istradefylline on levodopa dose titration are limited. The ISTRA ADJUST PD study will evaluate the effect of adjunctive istradefylline on levodopa dosage titration in PD patients.

**Methods:**

This 37-week, multicenter, randomized, open-label, parallel-group controlled study in PD patients aged 30–84 years who are experiencing the wearing-off phenomenon despite receiving levodopa-containing medications ≥ 3 times daily (daily dose 300–400 mg) began in February 2019 and will continue until February 2022. Enrollment is planned to attain 100 evaluable patients for the efficacy analyses. Patients will receive adjunctive istradefylline (20 mg/day, increasing to 40 mg/day) or the control in a 1:1 ratio, stratified by age, levodopa equivalent dose, and presence/absence of dyskinesia. During the study, the levodopa dose will be increased according to symptom severity. The primary study endpoint is the comparison of the cumulative additional dose of levodopa-containing medications during the treatment period between the adjunctive istradefylline and control groups. Secondary endpoints include changes in efficacy rating scales and safety outcomes.

**Discussion:**

This study aims to clarify whether adjunctive istradefylline can reduce the cumulative additional dose of levodopa-containing medications in PD patients experiencing the wearing-off phenomenon, and lower the risk of levodopa-associated complications. It is anticipated that data from ISTRA ADJUST PD will help inform future clinical decision-making for patients with PD in the real-world setting.

**Trial registration:**

Japan Registry of Clinical Trials, jRCTs031180248; registered 12 March 2019.

## Background

Parkinson’s disease (PD) is a progressive neurodegenerative movement disorder. The mechanism of pathogenesis stems from the striatal deficiency of the neurotransmitter dopamine due to the degeneration of dopaminergic neurons in the substantia nigra pars compacta of the midbrain [1, 2]. The well-recognized clinical manifestations of PD commonly include tremor, rigidity, bradykinesia, akinesia, and postural instability [3].

Levodopa was introduced in the late 1960s for the treatment of PD [4]. Levodopa crosses the blood–brain barrier and is converted to dopamine, and is currently the most effective treatment and an essential drug throughout the clinical course of the disease [5]. However, motor complications, such as the wearing-off phenomenon and levodopa-induced dyskinesia, can be a challenge when treating PD patients with dopaminergic agents over extended durations, with up to 50% of patients developing complications within 5 years of starting treatment [6–9]. Studies have suggested that these complications may be related to the daily levodopa dosage [10, 11]. In order to constrain the need for levodopa dose increases, alternative or adjunctive treatments may be considered. Dopamine agonists are often added to treatment regimens [7, 12] but also have problematic side effects, such as impulse control disorders, sleepiness, and hallucinations [13] which must be considered. Moreover, since levodopa-sparing therapies such as dopamine agonists appear to be less effective than levodopa over time [14], there remains situations in which increasing the levodopa dose is the best option for patients. Thus, the current clinical recommendation is that the lowest possible levodopa dose should be used to balance the risk/benefit profile of treatment [10, 15]. However, adjusting the dose of levodopa must be done cautiously, to avoid causing adverse effects whilst attempting to control symptoms. Over time, it becomes difficult to adjust the dose of levodopa for symptom control without introducing adverse effects, particularly after the onset of the wearing-off phenomenon. Although deep brain stimulation and levodopa-carbidopa intestinal gel are known to be effective for treating the wearing-off phenomenon, they are recommended for patients with late-stage PD due to the need for surgical intervention with these treatments. No current adjunctive pharmacotherapy to enable levodopa dose adjustment has proven sufficient to achieve satisfactory clinical control for many early-stage patients after the onset of the wearing-off phenomenon [16].

The adenosine A_2A_ receptor has been identified as one possible non-dopaminergic target for novel PD drugs. This receptor is highly expressed in the striatum and in the external globus pallidus, particularly in striatopallidal medium spiny neurons which form the indirect pathway in the basal ganglia system that are important for the control of voluntary movement [17]. The adenosine A_2A_ receptor antagonist istradefylline suppresses the excessive activation of the striatopallidal medium spiny neurons that occurs in PD, normalizing basal ganglia function, and thus improving PD symptoms [18, 19]. The results from several double-blind trials [20–24], meta-analyses [25–27], and a post-marketing surveillance study [28] have shown that istradefylline improves OFF time in the treatment of advanced PD. Istradefylline is currently available in Japan and the United States and is indicated as an adjunctive treatment to the levodopa-containing preparations in PD patients experiencing the wearing-off phenomenon [29].

Preclinical and clinical studies have suggested that adjunctive istradefylline may provide sustained antiparkinsonian benefits without a levodopa dose increase [30–32]. In primate studies, administration of istradefylline in conjunction with low-dose levodopa or with threshold-dose levodopa plus threshold-dose dopamine agonists was found to significantly improve motor function [31, 33]. A small-scale (*n* = 15), double-blind, placebo-controlled study conducted in the United States reported that adjunctive istradefylline (40 mg/day) administered with threshold-dose levodopa significantly improved motor function (measured using the Unified Parkinson’s Disease Rating Scale [UPDRS] part III score) [30]. In Japan, a small (*n* = 15) retrospective clinical study demonstrated that istradefylline might enhance the effects of low-dose levodopa and improve motor symptoms [32]. Furthermore, data from an interim analysis of a post-marketing surveillance study showed that in the real-world setting, where physicians are able to optimize treatment at their discretion, the dosage of levodopa in patients receiving istradefylline did not change significantly over a one-year period [28]. However, robust clinical data from large, well-designed prospective trials on the effect of istradefylline on levodopa sparing are lacking. Therefore, the objective of this multicenter, randomized, open-label, parallel-group controlled study (ISTRA ADJUST PD) is to evaluate the effect of adjunctive istradefylline on the cumulative dose of levodopa-containing products in patients with PD. This design was determined by the fact that simply reducing the levodopa dosage to directly evaluate levodopa-sparing effects is not ethically feasible. This assessment of whether istradefylline can suppress levodopa dose increases will provide indirect evidence for its putative levodopa-sparing effect. The efficacy and safety of istradefylline will also be evaluated. In this report, we describe the protocol and design rationale of the ISTRA ADJUST PD study.

## Methods/Design

### Patients

Patients eligible for inclusion in the ISTRA ADJUST PD study will be those with PD who meet all of the inclusion criteria and do not meet any of the exclusion criteria. Inclusion criteria include taking levodopa-containing medications ≥ 3 times daily with a daily dose of 300–400 mg; experiencing wearing-off phenomenon; age 30–84 years; PD diagnosed according to The International Parkinson and Movement Disorder Society (MDS) criteria [34]; modified Hoehn & Yahr (mH&Y) scale [35] (ON) stage ≤ 3; and provided written informed consent. Patients concomitantly taking anti-PD drugs other than levodopa, such as dopamine agonists, catechol-o-methyl-transferase inhibitors, and monoamine oxidase type B inhibitors, will also be eligible for inclusion. Exclusion criteria include previous treatment with istradefylline; receipt of any investigational drug within 4 months before the date of enrolment; presence of dementia or a Japanese version of the Mini-Mental State Examination (MMSE) score of ≤ 23; prior neurosurgery for PD (e.g., stereotactic surgery, deep brain stimulation, gamma knife); current or planned treatment with levodopa/carbidopa hydrate enteral suspension; moderate or severe hepatic disorder; PD treatment initiation or regimen change within 4 weeks before enrolment; receipt of strong CYP3A4 inhibitors (e.g., itraconazole, clarithromycin) within 14 days before enrolment; lactation or pregnancy; and any other reason for ineligibility according to the discretion of the investigator.

### Study design, treatments, and blinding

**Fig. 1:**
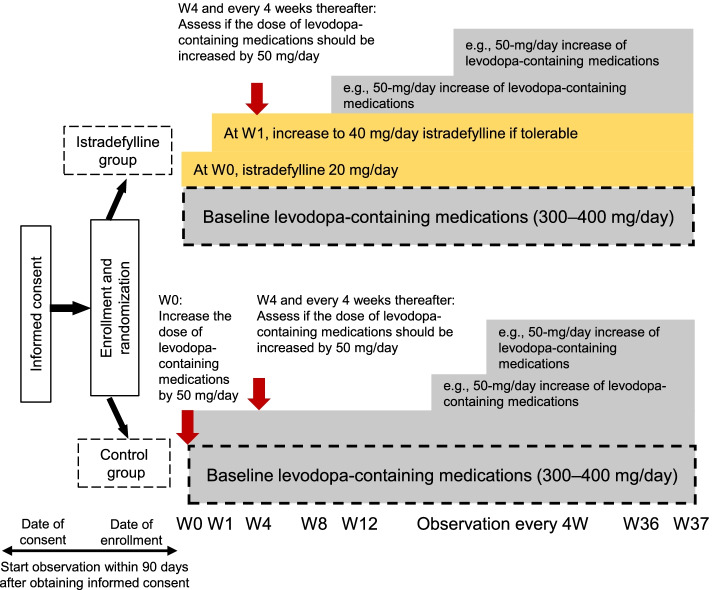
ISTRA ADJUST PD study design. W, week

The ISTRA ADJUST PD study is a multicenter, randomized, open-label, parallel-group controlled study (Fig. 1). Study participants will be randomly assigned to either the istradefylline group or the control group in a 1:1 ratio. Randomization will be centrally performed via computer allocation, using a minimization method and stratified according to age (< 60 or ≥ 60 years), levodopa equivalent dose (< 400 or ≥ 400 mg/day), and dyskinesia (presence or absence). The study will be conducted from February 2019 to February 2022 (with the registration period lasting until November 2020), and the expected duration of involvement for each participant is 37 weeks. The observation schedule is shown in Table 1; most observations will be conducted at 4- or 12-weekly intervals during the study period.

**Table 1:** Observation schedule

	Date of consent or enrollment	Observation period (week)											
		0	1^a^	4	8	12	16	20	24	28	32	36^b^	37^c^
Informed consent	●												
Eligibility confirmation	●	●											
MMSE	●												
Patient background	●												
Medical history/complications		●											
Decision on dose increase of levodopa-containing medications				●	●	●	●	●	●	●	●	●	
CGI-S		●		●	●	●	●	●	●	●	●	●	
CGI-I				●	●	●	●	●	●	●	●	●	
PGI-S		●		●	●	●	●	●	●	●	●	●	
PGI-I				●	●	●	●	●	●	●	●	●	
mH&Y (ON/OFF)		●				●			●			●	
MDS-UPDRS (part I, II, III, IV)		●				●			●			●	
PDQ-39		●				●			●			●	
Wearable device data	●					●			●			●^e^	
Symptom diary^d^	●					●			●			●^e^	
Hospitalizations	●	●	●	●	●	●	●	●	●	●	●	●	●
Concomitant medication check	●	●	●	●	●	●	●	●	●	●	●	●	●
Adverse events	●	●	●	●	●	●	●	●	●	●	●	●	●
Information on discontinuation		At any time											
Data entry into the case report form		Within 2 weeks after each visit											

*CGI-I* clinical global impressions of improvement, *CGI-S* clinical global impressions of severity, *MDS-UPDRS* Movement Disorder Society Unified Parkinson’s Disease Rating Scale, *mH&Y* modified Hoehn & Yahr scale, *MMSE* Mini-Mental State Examination, *PDQ* Parkinson’s disease questionnaire, *PGI-I *patient global impressions of improvement, *PGI-S* patient global impressions of severity

^a^Istradefylline group only

^b^Or the day immediately after discontinuation

^c^Not a mandated visit if judged to be unnecessary

^d^Symptoms will be recorded in symptom diaries by study participants or their family members/caregivers. The investigator, subinvestigator, or study cooperator are also allowed to record symptoms in the diary during the visit, or in the case that the patients are hospitalized. Visits will be conducted in an outpatient setting; however, inpatient visits will also be allowed if needed

^e^Wearable devices will be worn for one additional week after the end of the observation period (from Week 36), and symptoms are also recorded in the diary during this period; if the patient has discontinued, these recordings are terminated on the day of discontinuation

The criteria for the titration of levodopa-containing medications are as follows: if the clinical global impression of severity scale (CGI-S) is ≥ 4 on the day of observation, the dose is incrementally increased by 50 mg/day. If an intolerable adverse reaction occurs due to the dose increase, reduction of the dose of the levodopa-containing medication is allowed (at the physician’s discretion; no reduction amount is specified). For levodopa, only a dose increase of 50 mg/day per visit will be allowed.

Once-daily oral administration of a 20 mg istradefylline tablet will be started at Week 0. At Week 1, if the treatment is well tolerated and motor symptoms persist, the dosage of istradefylline will be increased to 40 mg, once daily. Dose reduction is allowed if treatment is not tolerable.

Changes in the dosage and dosing regimen of anti-PD drugs are not permitted within 4 weeks before enrollment and during the treatment period. However, dose reduction of a specific drug is permitted, if intolerable adverse reactions causally associated with that drug appear.

### Efficacy outcomes

The primary study endpoint is the comparison of the cumulative additional dose of levodopa-containing medications during the treatment period (from Week 0 to Week 37) between patients with PD who receive adjunctive istradefylline and those who do not.

Secondary efficacy endpoints are the between-group comparisons of additional levodopa doses on each observation day from Week 4 to Week 36; the number of days until the first dose increase after Week 4; change in dose of levodopa-containing medications until Week 36; CGI-S score and change in score; CGI-improvement scale (CGI-I) score (to measure the degree of improvement from the previous evaluation); patient global impression of severity (PGI-S) score and change in score; PGI-improvement (PGI-I) score (to measure the degree of improvement from the previous evaluation); mH&Y staging scale (ON/OFF) score and change in score; MDS-UPDRS part I score (nonmotor experiences of daily living), part II score (motor experiences of daily living), part III score (motor examination), part IV score (motor complications), and change in scores; Parkinson’s Disease Questionnaire-39 (PDQ-39) score and change in score; and correlations between any of the score changes. Correlations between CGI-S and other measures (e.g., MDS-UPDRS3, MDS-UPDRS4, and PGI-S) will be evaluated to confirm whether dose escalation decisions would differ between using CGI-S and using other measures.

Patients will also wear a wristband-type triaxial accelerometry system (UW-301BT, Hitachi Systems Ltd., Tokyo, Japan) on their non-dominant wrist for 7 days every 3 months during the study; data extracted from the wearable device will include movement frequency and intensity, gait (step count, pace, and laterality), and sleep (bedtime, awakening time, duration of sleep, sleep efficiency, sleep onset latency, movement during sleep, and number of episodes of getting out of bed).

Safety endpoints for this study will include adverse events and adverse drug reactions classified based on System Organ Class and Preferred Term defined in the Medical Dictionary for Regulatory Activities (Japan edition), using the most recent version at the time of coding.

Exploratory endpoints include evaluation of the correlation between the information extracted from the wearable device and the primary/secondary efficacy endpoints.

### Statistical methods

There have been no previous studies investigating the effect of istradefylline treatment on the flexibility of the doses of levodopa-containing formulations. Thus, we used real-world data based on information from the medical claims database (Medical Data Vision Co., Ltd., Tokyo, Japan) to estimate the between-group difference in the Mann–Whitney U test by simulating cumulative L-DOPA doses in PD patients (non-istradefylline treatment), which would ensure a power of 80% with groups of 50 patients. In this condition, an approximate 21.3% in between-group difference over a period of 9 months (270 days) is detectable as the area under the curve (AUC) of cumulative additional L-DOPA doses between treatments (data on file; Kyowa Kirin Co., Ltd., Tokyo, Japan). Based on the mean additional levodopa dose over 9 months (approximately 265 mg) in a previously published study [36], recommendation from the Expert Medical Advisory board for this study, and the clinical experience in Japan, we anticipated that istradefylline might suppress the dose increase of levodopa-containing medications by 20%. Therefore, the sample size was set at 111 patients, to allow for 10% drop-outs and to ensure that there were 50 patients per group with data evaluable for the efficacy analyses.

The efficacy and safety analysis sets will include all patients who meet the inclusion criteria and do not meet the exclusion criteria, with the exception of those who withdraw consent before the start of the observation period (Week 0) or are withdrawn by the study investigator, and those randomly assigned to istradefylline treatment but who do not start administration of istradefylline. The patient background data that will be collected will include date of birth (if not disclosed, age at the date of consent), sex, height, weight, caregiver availability, pregnancy, hospitalization, year of onset of PD, year of onset of wearing-off, family history of PD, year of initial treatment with levodopa, daily dose and number of administrations of levodopa, levodopa equivalent dose, MMSE score, and mH&Y (ON/OFF).

Summary statistics will be calculated for quantitative variables, and the number of patients and percentage will be determined for categorical variables. For the primary endpoint, the cumulative additional levodopa dose (AUC during the treatment period) will be calculated using the Mann–Whitney U test. The cumulative additional dose (AUC of additional dose during the treatment period) will be calculated as the total dose (daily dose × number of days) added to the dose of levodopa-containing medications (300–400 mg/day) at randomization. For the secondary endpoints, the cumulative additional doses of levodopa will be calculated for the patients who completed the treatment (Week 37). A log-rank test will be performed on the number of days from the start of observation (Week 0) to the time of dose increase, and time-to-event curves will be created using the Kaplan–Meier method. Summary statistics of the secondary endpoints at each observation time point will be calculated for each group, and mean and standard deviation data will be plotted in a line graph. Scores at each time point will be compared between groups using the Mann–Whitney U test. Correlation of the secondary endpoint scores will be performed using the Spearman correlation coefficient, and scatter plots will be created. A two-sided significance level of 5% will be applied to between-group comparisons, and two-sided 95% confidence intervals will be calculated. No imputation will be made for missing data. We will prepare a detailed statistical analysis plan before the database is finalized and locked. Statistical analyses will be conducted using SAS version 9.4 (SAS Institute Inc., Cary, NC, USA).

## Discussion

The ISTRA ADJUST PD randomized, controlled, open-label study is designed to address several key questions in the treatment of PD patients. At the end of the study, the study data will be published in academic meetings or journals in Japan and globally, and it is expected that the results will add to the available knowledge base and help to inform future clinical decision-making. It is imperative that clinicians have access to robust data examining the impact of different treatment options in patients experiencing the wearing-off phenomenon. Primarily, our study intends to identify whether the administration of istradefylline has any effect on the cumulative additional dose of levodopa-containing medications, thereby providing indirect evidence for levodopa-sparing. In addition to the treatment effect of istradefylline on the symptoms of wearing-off, we will also examine whether the administration of adjunctive istradefylline has any effect on the final dose of levodopa-containing medications; this will help to inform future treatment decisions for PD patients using istradefylline.

Previous publications have suggested that the daily dose of levodopa may be predictive of treatment-induced dyskinesia [11, 37–39], with the risk of motor complications increasing with doses above 400 mg/day [10]. Therefore, in this study, we will evaluate the effect of istradefylline on dose escalation from the daily levodopa dose of 300–400 mg, which requires careful judgment. We anticipate that the results obtained will be of immense practical use to physicians in assessing routine clinical issues associated with dose escalation above 400 mg/day. As the clinical benefits and adverse effects may be difficult to balance in patients with advanced PD, it is considered necessary that the levodopa dose modifications are made in small increments; thus, each dose escalation in this study was set to 50 mg.

There is considerable interest in a personalized approach to PD treatment [40–42], largely due to the clinical heterogeneity observed amongst patients. However, for this approach to be successful, there must be a clearer understanding of the associations between different subjective and objective measures of PD symptoms and the therapeutic outcomes. In this study, we intend to evaluate whether there is any relationship between the change in the index used to determine the dose increase of levodopa-containing medications (CGI-S) and the change in other indicators (CGI-I, PGI-I, PGI-S, CGI-S, mH&Y, MDS-UPDRS parts I–IV, PDQ-39, and data from the wearable device). The CGI was chosen as the criterion for increasing the levodopa dose as this reflects the routine clinical measure by which physicians judge dose adjustment; thus, by investigating the correlation between CGI and the other specified indicators, we can ascertain the objectivity of the CGI as a tool for therapeutic decision-making.

Finally, more severe dyskinesia is known to have a negative impact on patient quality of life (QOL) [43–45]. By identifying a therapy that reduces the levodopa dose and the risk of complications associated with levodopa-containing medications, studies such as this can contribute considerably to improving the QOL of PD patients and their families/caregivers. We believe that this study will provide a much-needed source of additional information, which can be used to underpin future treatment recommendations and practice to improve not only objective motor and non-motor symptoms, but also subjective and objective QOL, for the benefit of real-world PD patients.

### Limitations

We acknowledge that our study has several limitations, of which the most notable is the open-label design. However, the inclusion of objective measures using the wearable device is intended to offset any bias resulting from the open-label study design and unblinded knowledge of the drug regimen administered. Moreover, the study aims to evaluate the levodopa-sparing effect; however, due to the ethical feasibility issue for patients, the study is designed to evaluate additional levodopa dosage. We also note that any conclusions resulting from our analysis must be made with caution, since we are unable to exclude the effects of PD drugs other than levodopa, and the short (37-week) observation period precludes long-term evaluations of effects in this chronic disorder. Because of the heterogeneity of PD and the small number of study participants, there is a risk of bias as some subtypes may be over-represented in one group over the other. The average timing for additional administration of levodopa remains unclear; thus, a 37-week observational period might not be sufficient to detect changes in levodopa requirements. However, in a previous randomized controlled study [36], the levodopa dose at 9 months increased to approximately 265 mg. This was a levodopa-controlled, flexible-dose study, similar to the current study, and the average levodopa doses at baseline between the studies are comparable. Therefore, we believe that the 37-week observation period will be enough to detect changes in levodopa requirements. Finally, we recognize that the lack of a pathologically confirmed diagnosis may lower the accuracy of the PD diagnosis. Nonetheless, we feel that any confounding effect is likely to be small, given that eligible patients must have had PD diagnosed using standardized, international MDS criteria and have been receiving treatment for PD prior to enrolment.

## Conclusions

In this 37-week study, we aim to clarify whether adjunctive istradefylline can reduce the cumulative additional dose of levodopa-containing medications in PD patients experiencing wearing-off phenomenon. Changes in symptoms and safety will also be assessed, and we anticipate that the resulting data will expand our understanding of how PD patients experiencing the wearing-off phenomenon may be treated in order to minimize the risk of unwanted levodopa-related motor complications.

## Data Availability

There are no data associated with the current paper, which describes a protocol for a clinical trial that is in progress at the time of submission. All investigators will have access to the final dataset.

## Abbreviations

AUC: Area under the curve
CGI-I: Clinical global impressions of improvement
CGI-S: Clinical global impressions of severity
MDS-UPDRS: Movement Disorder Society Unified Parkinson’s Disease Rating Scale
mH&Y: Modified Hoehn & Yahr Scale
MMSE: Mini-Mental State Examination
PD: Parkinson’s Disease
PDQ: Parkinson’s Disease Questionnaire
PGI-I: Patient global impressions of improvement
PGI-S: Patient global impressions of severity
QOL: Quality of life
